# Biomarkers of Pediatric Brain Tumors

**DOI:** 10.3389/fped.2013.00007

**Published:** 2013-03-21

**Authors:** Mark D. Russell, Adam M. H. Young, Surya K. Karri

**Affiliations:** ^1^School of Clinical Medicine, Addenbrooke’s Hospital, University of CambridgeCambridge, UK; ^2^Department of Neurosurgery, Massachusetts General Hospital, Harvard Medical School, Harvard UniversityBoston, MA, USA

**Keywords:** biomarker, pediatric, brain, tumor, CNS, marker, medulloblastoma

## Abstract

**Background and Need for Novel Biomarkers:** Brain tumors are the leading cause of death by solid tumors in children. Although improvements have been made in their radiological detection and treatment, our capacity to promptly diagnose pediatric brain tumors in their early stages remains limited. This contrasts several other cancers where serum biomarkers such as cancer antigen (CA) 19-9 and CA 125 facilitate early diagnosis and treatment.

**Aim:** The aim of this article is to review the latest literature and highlight biomarkers which may be of clinical use in the common types of primary pediatric brain tumor.

**Methods:** A PubMed search was performed to identify studies reporting biomarkers in the bodily fluids of pediatric patients with brain tumors. Details regarding the sample type [serum, cerebrospinal fluid (CSF), or urine], biomarkers analyzed, methodology, tumor type, and statistical significance were recorded.

**Results:** A total of 12 manuscripts reporting 19 biomarkers in 367 patients vs. 397 controls were identified in the literature. Of the 19 biomarkers identified, 12 were isolated from CSF, 2 from serum, 3 from urine, and 2 from multiple bodily fluids. All but one study reported statistically significant differences in biomarker expression between patient and control groups.

**Conclusion:** This review identifies a panel of novel biomarkers for pediatric brain tumors. It provides a platform for the further studies necessary to validate these biomarkers and, in addition, highlights several techniques through which new biomarkers can be discovered.

## Introduction

### Background

Brain tumors are the leading cause of death from solid tumors in children (1). This statistic, coupled with the rising incidence rate of pediatric brain tumors from 2.3 cases per 100,000 in 1975 to 3.6 cases per 100,000 in 2009 (1), highlights the need for novel approaches aimed at tackling these tumors.

Whereas serum biomarkers, such as cancer antigen (CA) 125 and CA 19-9, facilitate the early diagnosis and treatment of several cancers of adults, few biomarkers exist for pediatric malignancies. Examples include alpha-fetoprotein in hepatoblastomas and yolk sac tumors, and neuron-specific enolase in neuroblastomas. Biomarkers of pediatric brain tumors remain elusive however, with the exception of alpha-fetoprotein and β-human chorionic gonadotropin in intracranial germ cell tumors (2, 3).

### Biomarkers and their clinical use

A tumor biomarker is a substance, measureable in cancer patients, whose detection reflects the presence of a tumor (4). One clinical use for tumor biomarkers is in the screening of asymptomatic populations for occult tumors. Despite a rising incidence rate, pediatric brain tumors remain relatively uncommon. As such, mass screening of asymptomatic children using novel biomarkers is unlikely to be cost-effective at present. Fluid analysis for novel biomarkers could, however, facilitate the timely diagnosis of children in whom there is a clinical suspicion of a brain neoplasm. Furthermore, an early histological diagnosis may be made possible by analysis for biomarkers specific to particular types of pediatric brain tumor. This would help direct pre-operative management, such as chemotherapy and radiotherapy, as well as aid prognostication. Following treatment, serial measurement of biomarker levels could also be used to monitor treatment response and detect recurrence.

### Current challenges

A likely contributing factor to the paucity of biomarkers in the serum of pediatric brain tumor patients is the blood-brain barrier. The cerebrospinal fluid (CSF) has thus been investigated as an alternative diagnostic fluid to serum in the search for pediatric brain tumor markers. Numerous CSF biomarkers of pediatric brain tumors were highlighted in literature reviews published in the 1980s (5). Perhaps the most promising were polyamines in children with medulloblastomas. One study demonstrated consistent elevations in CSF levels of putrescine, a polyamine, in 32 children with medulloblastomas relative to controls (6). Many other candidates were too non-specific for routine clinical use, however.

Moreover, the value of urine as an alternative diagnostic fluid to serum and CSF is already well established in the case of one pediatric malignancy. In neuroblastomas, quantification of urinary catecholamine metabolites facilitates disease diagnosis, with a reported specificity and sensitivity of >99 and 66–100%, respectively (7).

### Aims

The search for biomarkers in the serum, CSF, and urine of pediatric brain tumor patients has continued in recent years. We review the latest literature and highlight those biomarkers which may be of clinical use in the common types of primary pediatric brain tumor. By identifying such biomarkers we hope to facilitate the prompt diagnosis and treatment of novel and recurrent disease through minimally invasive means.

## Methods

A PubMed search was performed to identify studies reporting biomarkers in the prevalent forms of primary pediatric brain tumor. The following terms were combined along with appropriate synonyms, abbreviations, and MeSH terms: “serum,” “plasma,” “blood,” “CSF,” “urine,” “biomarker,” “marker,” “pediatric,” “brain,” “central nervous system (CNS),” “tumor,” “neoplasm,” “medulloblastoma,” “glioma,” “pilocytic,” “astrocytoma,” “primitive neuroectodermal,” “craniopharyngioma,” “atypical teratoid,” “ependymoma.”

The search was limited to manuscripts published since 1990. Studies reporting biomarkers in the serum, CSF, and urine of pediatric brain tumor patients that were differentially expressed relative to controls were selected. For each study selected, the number and age of patients and controls was recorded along with details regarding the biomarkers being analyzed, the diagnostic fluid chosen, patient tumor types, quantification methodologies, and any outcome measures reported.

## Results

Twelve studies were identified, highlighting a total of 19 biomarkers in 367 tumor patients vs. 397 controls (Table 1). The biomarkers from all but one study were able to discriminate between tumor patients and controls to a statistical significance of *p* ≤ 0.05. In the remaining study, the authors did not specify the statistical significance of their findings (8).

**Table 1:** **Reports in the literature describing fluid biomarkers of pediatric brain tumors**.

Authors	Year	Biomarker(s)	Sample type(s)	Methodology	*n*	Tumor type(s)	Findings	*p* Value
Smith et al. (10)	2008	MMP-2,-9,-9/NGAL and VEGF	Urine, CSF	ELISA, zymography	51	Various CNS tumors	Elevations in urinary MMP-2, -9, -9/NGAL and VEGF in tumor patients. MMP-9 elevated in CSF of tumor patients	<0.001 (Urine), <0.05 (CSF)
de Bont et al. (18)	2006	Apolipoprotein A-II	CSF	MS, WB	102	Various CNS tumors	Overexpressed in tumor patients	<0.0000001
Li et al. (19)	1994	bFGF	CSF	ELISA	44	Various CNS tumors	Detectable in 16/26 tumor patients, but not in controls	<0.0001
Rajagopal et al. (20)	2011	PGD2S, Apo E and J	CSF	2D-electrophoresis, MS, ELISA	58	Medullo.	PGD2S and Apo E reduced, and Apo J increased, in tumor patients	<0.00001 (PGD2S), <0.01 (Apo E and J)
de Bont et al. (22)	2008	IGFBP-3,4,6	Blood, CSF	Radioimmune assay	62	Medullo., ependy.	IGFBP-3, -4, -6 elevated in the CSF, but not blood, of medulloblastoma patients	<0.001/ = 0.011/ = 0.010
Müller et al. (23)	1993	IGFBP-3	Serum, CSF	Radioimmune assay, WB	61	Various CNS tumors	Elevated in CSF, but not serum, of 16/23 tumor patients, incl. 12/13 medullo./ependy. patients	<0.0001
Müller et al. (24)	1994	IGFBP-2	Serum, CSF	Radioimmune assay, WB, IP	67	Various CNS tumors	Elevated in CSF, but not serum, of tumor patients	<0.001
Figarella-Branger et al. (28)	1996	PSA-NCAM	CSF	ELISA	43	Medullo.	Concentrations greater in treatment-refractory and relapsing patients than patients in remission or controls	<0.05
de Bont et al. (29)	2008	t-Tau, p-Tau(181P)	CSF	IP	61	Various CNS tumors	t-Tau and p-Tau(181P) increased and decreased in tumor patients, respectively	<0.001 ( = 0.002 for t-Tau in non-medullo.)
Kao et al. (35)	2005	Osteopontin	Plasma, CSF	ELISA	39	AT/RT, medullo.	Elevated in the CSF and plasma of AT/RT patients	<0.05
Krizkova et al. (9)	2010	Metallothionein	Serum	DVP, WB	96	Solid tumors	Elevated in cancer patients’ sera, incl. that of medullo. and ependy. patients	<0.05
Behrends et al. (8)	2003	Humoral responses	Serum	cDNA libraries, plaque assays	80	Various cancers	Humoral responses to several medullo. antigens noted exclusively/predominantly in cancer patients.	Not specified

*See text for individual details. *p* Value significant if *p* < 0.05. WB, western blot; MS, mass spectrometry; IP, immunoprecipitation; DVP, differential pulse voltammetry; ELISA, enzyme-linked immunosorbent assay*.

Of the 19 biomarkers reported, 12 were biomarkers in CSF alone, 2 in serum alone, 3 in urine alone, 1 in CSF and plasma, and 1 in CSF and urine. A wide range of techniques were used to detect and quantify the biomarkers, with enzyme-linked immunosorbent assays (ELISA), western blots, and radioimmune assays being the most frequently used. The majority of studies included a variety of CNS tumors in patient cohorts. Only two studies included tumors from outside the CNS within the patient cohort (8, 9). In both cases, the authors separated these tumors from those of CNS origin during subsequent analysis.

In all but one study, patient cohorts were of a pediatric/young adult age range. The remaining study included both adult and pediatric patients but proceeded to analyze the two age groups separately (10). The control cohort used in this study also included both children and adults, but again, these groups were separated during further analysis. All other studies used only controls of a young age, apart from one study which compared pediatric tumor patients to healthy adult controls (9).

## Discussion

### Urinary matrix metalloproteinases

Matrix metalloproteinases (MMPs) are degradative enzymes with central roles in tumor neovascularization, invasion, and metastasis. Several MMPs are known to be overexpressed in primary brain tumors (11). This, coupled with the detection of MMPs in the urine of patients with a variety of cancers (12–15) prompted the investigation of their utility as urinary biomarkers of brain tumors (10). In this study, MMP-2, MMP-9, and MMP-9/NGAL, in addition to the pro-angiogenic molecule vascular endothelial growth factor (VEGF), were found to be elevated in the urine of 28 brain tumor patients relative to controls (*p* < 0.001). Although the cohort of brain tumor patients contained both children and adults, the elevations remained statistically significant on analysis of the 11 pediatric patients alone. With optimal cut-off levels, MMP-2 and VEGF had sensitivities of 82.1 and 95.2% and specificities of 95.7 and 89.5%, respectively, in differentiating tumor patients from controls.

During the longitudinal follow-up of a subset of patients, the authors observed clearance of the biomarkers following tumor resection. When coupled with their demonstration of a correlation between overexpression of MMPs and VEGF in tumor specimens and their presence in urine, these findings suggest a tumor-source for the urinary biomarkers.

The authors also noted elevations of MMP-9 in the CSF of brain tumor patients relative to controls (*p* < 0.05). This is in keeping with the results of a previous study, which detected MMP-9 in the CSF of 10/15 adult brain tumor patients with positive CSF cytology, compared to 0/27 controls (16). Interestingly, this study also found pro-MMP-9, the precursor of MMP-9, to be detectable in the CSF of all 39 brain tumor patients irrespective of CSF cytology status but in none of the controls. The potential utility of MMP-9 as a biomarker in urine and CSF contrasts the results of a recent study investigating MMP-9’s role as a serum biomarker of gliomas (17). In this study, the longitudinal monitoring of serum MMP-9 concentrations was not found to correlate with survival or disease status.

### Apolipoprotein A-II

SELDI-TOF mass spectrometry was used by de Bont et al. to compare the protein expression profiles of CSF samples obtained from 32 pediatric patients with a wide range of brain tumors and 70 pediatric controls (18). Of the protein peak clusters found to be differentially expressed between the two groups, apolipoprotein A-II was found to be highly significant (*p* < 0.0001) and double-loop classification analysis of the differentially expressed protein clusters demonstrated a mean prediction accuracy and specificity of 88% in separating brain tumor patients from controls. The high CSF/blood albumin concentration ratio and lack of cellular apolipoprotein A-II staining in tumor patients led the authors to conclude that the elevated apolipoprotein A-II levels resulted from entry from blood into the CSF across a leaky blood-brain barrier as opposed to increased tumor production.

### Basic fibroblast growth factor

In view of the abundant neovascularization present in brain tumors, one group analyzed levels of basic fibroblast growth factor (bFGF), a pro-angiogenic molecule, in the CSF of 26 pediatric and adolescent brain tumor patients (19). bFGF was found to be detectable in 16 of the 26 brain tumor patients but no controls (*p* < 0.0001). Furthermore, the concentration of bFGF in CSF samples correlated with the microvessel density of tumor sections from the same patients (*p* < 0.005), which, in turn, was associated with tumor recurrence (*p* = 0.005) and mortality (*p* = 0.02). The authors concluded that bFGF may be an important angiogenic mediator in brain tumors and thus a novel therapeutic target, as well as a diagnostic and prognostic biomarker.

### Prostaglandin D2 synthase and apolipoproteins E and J

In a recent study, CSF samples from 33 children with medulloblastomas were analyzed using two-dimensional electrophoresis and mass spectrometry (20). This demonstrated a sixfold mean reduction in prostaglandin D2 synthase (PGD2S) levels in medulloblastoma patients relative to age-matched controls (*p* < 0.00001). Similarly, the authors reported a nearly eightfold reduction in PGD2S concentration in tumor patients when quantified using ELISA (*p* < 0.000002). The CSF levels of apolipoproteins E and J were also noted to be different in medulloblastoma patients relative to controls, albeit less significantly, with a twofold reduction and three-fold elevation, respectively (*p* < 0.01 in both cases).

### Insulin-like growth factor pathway

In view of the likely role played by the insulin-like growth factor (IGF) system in the development of CNS neoplasms (21), several studies have investigated their utility as biomarkers of pediatric brain tumors.

In one study, radioimmune assays were used to determine the protein levels of various components of the IGF pathway in the blood and CSF of children with medulloblastomas and ependymomas (22). Insulin-like growth factor-binding protein-3 (IGFBP-3) showed most promise as a biomarker. Relative to age- and gender-matched controls, median levels of IGFBP-3 were significantly higher in the CSF, but not blood, of children with medulloblastomas (*p* < 0.001). Protein levels of IGFBP-4 and IGFBP-6 were also noted to be significantly elevated in the CSF of medulloblastoma patients (*p* = 0.011 and *p* = 0.010, respectively). When corrected for total CSF protein content, however, only IGFBP-3 elevations remained statistically significant relative to controls. The findings of de Bont et al. are in line with those of an earlier study, in which elevations of IGFBP-3 were found in the CSF, but not serum, of 16 out of 23 children with CNS neoplasms (*p* < 0.0001) (23).

A separate study by Müller et al. investigated IGFBP-2 as a biomarker in 21 children with malignant CNS tumors (24). IGFBP-2 was significantly elevated in the CSF, but not serum, of children with CNS tumors (*p* < 0.001), contrasting the findings of de Bont et al. who failed to show elevations in IGFBP-2 in the CSF of medulloblastoma and ependymoma patients (22). de Bont et al. note, however, that Müller et al. failed to correct for total CSF protein count and thus a non-specific elevation of protein in the CSF cannot be excluded.

### Polysialic-neural cell adhesion molecule

Polysialic-neural cell adhesion molecule (PSA-NCAM) is a cell-surface glycoprotein, transiently expressed in the developing brain but not in mature cells (25). PSA-NCAM is re-expressed in tumors of neuroectodermal origin, including medulloblastomas, and its presence in the CSF of several medulloblastoma patients appears to correlate with invasion of the meninges by the tumor (26, 27).

One study investigated PSA-NCAM’s role as a biomarker of meningeal invasion in the follow-up of patients with medulloblastoma (28). ELISA was used to quantify the concentration of PSA-NCAM in samples of CSF taken at various points in the follow-up of 29 children with medulloblastomas. In treatment-refractory and relapsing patients, PSA-NCAM concentrations were significantly higher than in patients in remission (*p* < 0.05), with concentrations undetectable in the CSF of controls. Of the eight medulloblastoma patients with persistently elevated PSA-NCAM levels all but one were refractory to treatment and all eight patients died during follow-up. Of the 12 medulloblastoma patients with persistently undetectable PSA-NCAM, 11 achieved first remission and lived to the end of the follow-up period. PSA-NCAM may therefore have a role in the staging, prognostication, and ultimately management of medulloblastomas.

### Tau

Tau is a group of microtubule-associated proteins found predominantly within neurons. Working on the hypothesis that neurodegeneration is likely to occur during brain tumor evolution, one group analyzed levels of total-Tau (t-Tau) and hyperphosphorylated-Tau_(181P)_ [p-Tau_(181P)_] in the CSF of 22 children with newly diagnosed brain tumors using monoclonal antibodies (29).

When compared to pediatric controls, t-Tau was significantly elevated in the CSF of children with both medulloblastomas (*p* < 0.001) and other types of brain tumor (*p* = 0.002); indicative of neuronal and axonal degeneration. Conversely, CSF levels of p-Tau_(181P)_ were significantly lower than controls, both in cases of medulloblastoma (*p* < 0.001) and other brain tumor types (*p* < 0.001). The authors speculate whether this reflects an increase in phosphatase activity in tumor patients.

### Osteopontin

Osteopontin is a bone matrix glycoprotein overexpressed in numerous cancers and implicated in several tumorigenic processes (30, 31), including via the induction of MMPs (32, 33).

Following their demonstration that the osteopontin gene and its protein product are up-regulated in primary cell cultures and tumor specimens from CNS atypical teratoid/rhabdoid tumors (AT/RTs) (34), Kao et al. investigated the osteopontin protein product as a fluid biomarker of CNS AT/RTs (35). Using ELISA, the median osteopontin levels were shown to be significantly elevated in the plasma and CSF of children with CNS AT/RTs, relative to medulloblastoma patients and controls (*p* < 0.05). Moreover, elevations in osteopontin correlated inversely with patient survival time (*p* < 0.0001, plasma; *p* = 0.0003, CSF). Further analysis of five AT/RT cases found that plasma osteopontin levels decline on tumor remission and increase on tumor relapse (*p* < 0.05). The authors went on to demonstrate amplification of osteopontin transcripts in AT/RT tumor specimens, supporting increased tumor production as the source of elevated osteopontin protein levels in the plasma and CSF.

### Metallothionein

Metallothioneins are a family of intracellular proteins implicated in several aspects of oncogenesis (36). In a recent study, metallothionein levels were quantified in the sera of 38 children with a variety of newly diagnosed solid tumors (including 10 medulloblastomas and 4 ependymomas) using differential pulse voltammetry (9). When compared to controls, a fivefold elevation in serum metallothionein levels was noted in children with cancer (*p* < 0.05). When analyzed separately, serum metallothionein levels were significantly elevated in both the medulloblastoma and ependymoma groups (*p* < 0.05). It should be noted, however, that the controls used in this study were healthy adults rather than children. Given the numerous metallothionein isoforms with variable tissue distributions, further investigation is needed to elucidate whether any isoforms in particular are elevated relative to others; as eluded to by the authors.

### Humoral responses to medulloblastoma antigens

Working on the principle that cancer patients may develop immune responses to tumor antigens, Behrends et al. investigated tumor-specific humoral responses as novel biomarkers of pediatric brain tumors (8). Using autologous sera, the authors screened the cDNA expression libraries of four medulloblastomas, highlighting 15 antigens of interest. Sera from 40 children with cancer, including 20 cancers of neuroectodermal origin, were then analyzed for humoral responses against each of the 15 antigens using plaque assays, and compared to healthy controls.

Humoral responses against five of the antigens were found exclusively in cancer patients, with frequencies ranging from 2/40 to 7/40 patients. Antibodies against two of these antigens were present in the sera of 2/5 medulloblastoma patients but in no controls. Two of the remaining 10 antigens had antibodies in the sera of 8/40 and 12/40 cancer patients but in only one control. This included 3/5 and 2/5 medulloblastoma patients, respectively, in addition to 1/2 astrocytoma patients in both cases. As the authors point out, immune responses against a homolog of the latter antigen, GLEA2, had previously been demonstrated in a study of glioma patients (37). In this study, anti-GLEA2 humoral responses were present in 20/50 glioma patients but in only 2/14 controls.

## Conclusion

### Summary

Brain tumors are the leading cause of solid tumor mortality in children. At present, no biomarkers exist for the prevalent forms of pediatric brain tumor. Their discovery could facilitate disease diagnosis, prognostication, management and follow-up, and may ultimately improve survival. Through a literature search, this study identifies 19 candidate biomarkers, all but one of which separated tumor patients from controls to a statistically significant degree.

The majority of these biomarkers were identified from single studies with widely varying cohorts and heterogeneous tumor types. Subsequently, there has been no apparent effort to validate these findings in second populations or to incorporate the biomarkers into prospective clinical trials. Although promising, it is not therefore possible to draw firm conclusions on the biomarkers’ clinical utility based on these studies alone.

This review highlights the pressing need for further study in a field that has had little study so far. Moreover, it provides a platform for subsequent study by identifying a panel of novel biomarkers warranting further investigation and an array of techniques through which this panel can be expanded upon.

### Future directions

Studies to validate the biomarkers presented here will require multi-center collaboration to increase cohort numbers and homogeneity. The separation of patient cohorts by tumor histology will help identify tumor type-specific biomarkers, facilitating early histological diagnoses and aiding both prognostication and pre-operative management.

Of note, several of the biomarkers identified here originate from processes such as blood-brain barrier disruption, neurodegeneration, extracellular matrix remodeling, and angiogenesis. These processes are by no means tumor-specific, and the inclusion of control cohorts such as stroke or trauma patients in subsequent studies will help determine the biomarkers’ specificity for tumors.

The choice of quantification technique and diagnostic fluid for further studies will need careful consideration of cost- and time-efficiency if the biomarkers are to enter the clinical arena. Urine is particularly appealing as a diagnostic fluid given the relative ease and low cost of its collection and analysis (10); especially true when compared to the expense and need for sedation in many pediatric imaging studies.

Novel avenues for biomarker discovery must be also investigated. A prime example is the recent detection of exosomes containing tumor-specific proteins, mRNA, and microRNAs in the sera of glioblastoma patients (38). As the authors state, nucleic acids carry great potential as biomarkers, both due to the availability of polymerase chain reaction as a highly sensitive means of detection and the presence of tumor-specific mutations such as EGFRvIII.

Finally, the application of multi-analyte profiling technology to the study of pediatric brain tumors could assist both biomarker discovery and validation. Multi-analyte profiling permits high-throughput screening of diagnostic fluids against large panels of biomarkers and has already advanced knowledge of biomarkers in a number of other conditions (39).

## Conflict of Interest Statement

The authors declare that the research was conducted in the absence of any commercial or financial relationships that could be construed as a potential conflict of interest.

## Abbreviations

AT/RT, atypical teratoid/rhabdoid tumor; bFGF, basic fibroblast growth factor; CNS, central nervous system; CSF, cerebrospinal fluid; ELISA, enzyme-linked immunosorbent assay; IGF, insulin-like growth factor; IGFBP, insulin-like growth factor-binding protein; MMP, matrix metalloproteinase; PGD2S, prostaglandin D2 synthase; PSA-NCAM, polysialic-neural cell adhesion molecule; VEGF, vascular endothelial growth factor.
